# Peritoneal antiseptic irrigation to prevent surgical site infection after laparotomy for hepatobiliary or gastrointestinal surgery (PAISI)—protocol for a randomized controlled study

**DOI:** 10.1186/s13063-022-06975-6

**Published:** 2022-12-20

**Authors:** Tara Mueller, Victoria Kehl, Silvia Egert-Schwender, Helmut Friess, Alexander Novotny, Daniel Reim

**Affiliations:** 1 Department of Surgery, Klinikum rechts der Isar, School of Medicine, Technical University of Munich, München, Germany; 2 Münchner Studienzentrum, School of Medicine, Technical University of Munich, München, Germany

**Keywords:** Surgical site infection, Abdominal surgery, Gastrointestinal surgery, Hepatobiliary surgery, Laparotomy, Peritoneal irrigation, Intracavity lavage, Sodium hypochlorite, Randomized controlled study

## Abstract

**Background:**

Postoperative surgical site infections (SSIs) remain common after laparotomy for resections of the gastrointestinal or hepatobiliary tract. Especially organ/space infections (CDC class III SSI) can be life-threatening, require relaparotomy, intensive care or interventional drainage of intraabdominal abscesses. The PAISI study aims to investigate whether the use of prophylactic peritoneal irrigation with NaOCl/HOCl solution can reduce the SSI rates following laparotomy for resections of the gastrointestinal or hepatobiliary tract, compared to standard irrigation with physiological electrolyte solution (Ringer’s solution). Secondarily, to evaluate whether the use of prophylactic peritoneal irrigation with NaOCl/HOCl solution can reduce postoperative morbidity and mortality as well as the rate of re-operations and length of hospital stay.

**Methods:**

PAISI is a prospective, randomized, observer- and patient-blinded, monocentric, two-arm surgical study in an adaptive parallel groups design, comparing peritoneal and wound irrigation with NaOCl/HOCl (50/50ppm) solution to irrigation with Ringer’s solution. The primary endpoint of the study is the SSI rate within 30 days postoperatively. Since there is no data on incidence rates from randomized clinical trials, the rates for sample size calculation were estimated according to the clinical experience at our institution. Therefore, the study design includes one unblinded look at the data by a second statistician, which will be performed after half of the patients reached the primary endpoint. This interim information will be used to check the assumptions and if needed, the sample size will be adjusted. The O’Brien-Fleming spending function is used to determine the efficacy test boundary and the non-binding futility boundary. The one-sided *z*-test (Group sequential test of two proportions) at the 2.5% significance level with a total of two looks at the data will have overall 80% power.

**Discussion:**

The results of this study will provide high-level evidence for future research and clinical recommendations regarding the use of NaOCl/HOCl solution in abdominal surgery and provide the participating patients the opportunity of a potentially improved treatment.

**Trial registration:**

German Clinical Trials Register (DRKS) DRKS00028037. Registered on 27 May 2022.

## Administrative information

The numbers in curly brackets in this protocol refer to SPIRIT checklist item numbers. The order of the items has been modified to group similar items (see http://www.equator-network.org/reporting-guidelines/spirit-2013-statement-defining-standard-protocol-items-for-clinical-trials/).

**Table:** 

Title {1}	Peritoneal antiseptic irrigation to prevent surgical site infection after laparotomy for hepatobiliary or gastrointestinal surgery (PAISI)
Trial registration {2a and 2b}.	The study was registered in the German Clinical Trials Register DRKS on the 27.05.2022, Nr.: DRKS00028037
Protocol version {3}	Version 3.0, 11.05.2022
Funding {4}	The study is supported by a grant from the Investigator Initiated Studies Program of Mölnlycke Healthcare
Author details {5a}	Dr. med. Tara Mueller, Prof. Dr. med. Daniel Reim, Prof. Dr. med. Alexander Novotny and Univ.-Prof. Dr. med. Helmut Friess, **Department of Surgery, Klinikum rechts der Isar, School of Medicine, Technical University of Munich, Germany** Dr. rer. nat. Victoria Kehl and Dr. rer. nat. Silvia Egert-Schwender, **Münchner Studienzentrum, School of Medicine, Technical University of Munich, Germany**
Name and contact information for the trial sponsor {5b}	In this study setting, investigating the effects of a routine medical procedure, there is no “trial sponsor”(according to German law). The study leadership consists of the principal initiating investigators: Dr. Tara Mueller and Prof. Daniel Reim Department of Surgery Klinikum rechts der Isar, Technical University of Munich Ismaningerstr. 22 81675 Munich, Germany Email: tara.mueller@tum.de; daniel.reim@tum.de
Role of sponsor {5c}	In this study setting there is no “trial sponsor” as outlined under {5b}. The study leadership has ultimate authority over study design; collection, management, analysis, and interpretation of data; writing of the report; and the decision to submit the report for publication.

## Introduction

### Background and rationale {6a}

Postoperative surgical site infection (SSI) is still one of the most common hospital infections. In Germany, approximately 200,000 SSIs are reported annually [1, 2]. In addition to the harm and discomfort for the patient, SSIs dramatically increase treatment costs and hospital occupancy. In Germany, postoperative SSIs account for approximately 1 million extra days of hospitalization and additional costs of around € 3 billion per year [3]. Other European countries report similar figures [4, 5]. According to the definition of the Centre for Disease Control and Prevention (CDC), SSI can be subdivided into 3 grades: class I (superficial wound infection), class II (deep wound infection) and class III (space/organ infection), see Table 1 [6]. While class I and II prolong the hospital stay and often require operative wound revision and long-term wound therapy, class III SSIs can be life-threatening and frequently require emergency relaparotomy, intensive care or interventional drainage of abscesses combined with systemic antibiotic therapy. Following open abdominal surgery with resections of the gastrointestinal or hepatobiliary tract, SSI rates remain especially high. Depending on the level of intraoperative contamination (LOC; see Table 2), recent high-level randomized controlled trials (RCTs) using standardized SSI definitions report postoperative SSI rates between 14.5 and 25.0% [8–10]. However, in those trials the incidence of SSIs class III is rarely reported separately and is thus difficult to estimate. In our institution, we observed an overall SSI rate of 13%, of which 7% were SSI class III, following elective laparotomies for any reason (LOC II–III) from 2013 to 2018 (unpublished data).

Prophylactic intraoperative peritoneal irrigation (IPI) of the abdominal cavity before closure of the fascia with physiological electrolyte or antiseptic solutions hypothetically represents an easy and economical option to reduce SSI rates and is already frequently used in clinical practice. A survey among 153 general surgeons showed that 97% perform IPI, while the frequency of use and choice of lavage fluid varied widely [11]. However, the latest official guidelines for the prevention of SSI by the CDC (2017) [12] and the World Health Organization (WHO, 2016) [5] conclude, that the use of prophylactic abdominal or intra-incisional irrigation represents an unresolved issue, as the available evidence suggests uncertain benefits [5, 12]. In contrast, the recently updated British guideline recommends not to use any intracavity or wound irrigation to prevent SSI due to insufficient evidence for its benefits [7, 13]. A large-scale Cochrane meta-analysis of intra-cavity and wound irrigation for prevention of SSI which included 59 RCTs with 14,738 participants (most of which focused on orthopaedic and trauma surgery) identified only 3 RCTs which reported intra-abdominal abscess formation as a separate outcome. They concluded that current evidence shows that there is no clear difference in the rate of abscess formation with or without IPI [14]. However, the trials included in the analysis are very heterogenous, as they assessed between irrigation and no irrigation, between antibacterial and non-antibacterial irrigation, between different antibiotics, different antiseptics, or non-antibacterial agents, and between different methods of irrigation delivery or volumes. In a recently published consensus on the use of antiseptic irrigation solutions for prophylaxis of SSIs, it is stated that for peritoneal rinsing, sodium hypochlorite solutions (NaOCl/HOCl) are the agent of choice [15]. The bactericidal mechanism is mediated through the ion OCl-, which is also formed during phagocytosis through enzyme mediation by myeloperoxidase, eosinophilic peroxidase, and superoxide dismutase [15, 16]. Pre-clinical studies, which were reviewed for the consensus showed that NaOCl/HOCl is highly effective against vegetative bacteria, bacterial spores, aspergilli, oocysts of cryptosporidia, coated viruses and against biofilm [15]. In addition, the speed of effect is superior to PVP-iodine, Octenidine and Polyhexanide. Due to its physiological mechanism of action, there is no evidence for any undesirable effects, cytotoxicity, or carcinogenic hazard [15].

#### Peritoneal lavage vs. no lavage

A meta-analysis of 6 RCTs (*n*=1633) from 2019 which compared IPI *vs.* suction alone during paediatric appendectomy for perforated appendicitis, showed no significant difference in the incidence of postoperative intraabdominal abscess formation, SSI, and length of hospitalization between the 2 groups. However, the evidence level of the included trials was low [17]. Another meta-analysis from the same year included only 3 RCTs and 5 retrospective analyses comparing suction alone *vs.* IPI to prevent intraabdominal abscess formation after appendectomy in adults. There was no evidence of benefit of IPI over suction for postoperative infective complications, and no individual study demonstrated a significant benefit in patients receiving IPI [18]. One RCT of IPI to prevent SSI after elective liver surgery in 193 patients showed that, when all grades of SSI were considered together, no significant difference was evident between lavage (21.9%) and non-lavage groups (13.4%, *p*=0.135). However, organ/space infection (SSI grade III) was significantly more frequent in the lavage group (16.7%) than in the non-lavage group (7.2%, *p*=0.048). Peritoneal lavage was even identified as a risk factor for organ/space infection by multivariate analysis [19]. Another RCT which investigated different volumes (5, 10 and 20 l) of IPI during emergency trauma laparotomy, found no differences with respect to infectious complications. However, the 20 l group showed a trend toward an increased incidence of deep SSIs when compared to the 5 and 10 l groups. However, this did not reach statistical significance [20].

#### Peritoneal lavage with NaOCl/HOCl vs. saline/ringer solutions

One clinical study evaluated the antimicrobial properties of NaOCl/HOCl solution when used for IPI in 110 patients undergoing emergency laparotomy for intestinal perforations. These patients were compared to a control group in which IPI with saline containing Metronidazole was used. The authors compared postoperative volume of drainage, drainage fluid culture, C-reactive protein (CRP) and Procalcitonin serum levels. They concluded that the use of NaOCl/HOCl for IPI was effective in reducing the risk of SSI and can be used safely to clean the operation site [21]. Another trial compared IPI with NaOCl/HOCl to saline in 44 paediatric patients with perforated appendicitis [22]. While no adverse effects were observed in the experimental group, the overall incidence of SSI was significantly reduced. However, there was no difference in the rate of abscess formation, duration of pyrexia, positive CRP, leucocytosis, or hospital stay between the groups. Another RCT of IPI with “super oxidized solution” (HOCl) compared to saline in 240 peritonitis cases in an Indian hospital showed a significant reduction of postoperative morbidity and mortality as well as SSI and pain in the intervention group [23]. Even though the literature evaluating SSI prevention is ever growing, high-level evidence regarding the prophylactic use of antiseptic IPI in abdominal surgery remains scarce and inconclusive due to the heterogeneity of trials.

### Objectives {7}

SSIs contribute significantly to postoperative morbidity and mortality. Clinical guidelines and clinical practice vary largely in terms of the use of peritoneal and wound irrigation to reduce the incidence of SSI [24]. The PAISI study aims to investigate if IPI with a NaOCl/HOCl solution can reduce the rate of postoperative SSI within 30 days (according to the CDC definition shown in Table 1) after resections of the gastrointestinal or hepatobiliary tract by laparotomy. The results of this study will provide high-level evidence for future research and clinical recommendations regarding the use of NaOCl/HOCl solution in abdominal surgery and provide the participating patients the opportunity of a potentially improved treatment.

**Table 1:** Definition and classification of surgical site infection (adapted from CDC) [6]

	Infection occurs within 30 days after the operation …
**Superficial incisional SSI** **(class I)**	**…and** infection involves **only skin or subcutaneous tissue** **and** at least ***one*** of the following: 1 Purulent drainage from the superficial incision 2 Organisms isolated from an aseptically obtained culture of fluid or tissue from the superficial incision. 3 At least one of the following signs or symptoms of infection: pain or tenderness, localized swelling, redness, or heat *and* superficial incision is deliberately opened by surgeon, *unless* incision is culture negative.
**Deep incisional SSI** **(class II)**	**…and** infection involves **deep soft tissues (fascial and muscle layers)** **and** at least *one* of the following: 1 Purulent drainage from the deep incision but not from the organ/space component of the surgical site. 2 A deep incision spontaneously dehisces or is deliberately opened by a surgeon 3 And at least one of the following symptoms: fever (>38°C), localized pain, or tenderness of the incision area *unless* incision is culture negative. 4 An abscess or other evidence of infection involving the deep incision is found on direct examination, during reoperation, or by histopathologic or radiologic examination.
**Organ/space SSI** **(class III)**	…**and** infection involves any part of the anatomy (e.g. organs or spaces), other than the incision, which was opened or manipulated during an operation **and** at least *one* of the following: 1 Purulent drainage from a drain that is placed through a stab wound into the organ/space. 2 Organisms isolated from an aseptically obtained culture of fluid or tissue in the organ/space. 3 An abscess or other evidence of infection involving the organ/space that is found on direct examination, during reoperation, or by histopathologic or radiologic examination.
	**…or the infection is diagnosed by the attending surgeon**

### Trial design {8}

This is a prospective, randomized, controlled, observer and patient-blinded, single-centre, two-arm surgical study with an adaptive parallel groups design. Pre-screening of potential patients (evaluation of inclusion and exclusion criteria) is possible up to 14 days prior to the planned procedure. Patients can be included if inclusion and exclusion criteria apply and written informed consent is provided. Included patients are randomized to peritoneal and epifascial wound irrigation with NaOCl/HOCl (50/50ppm) or Ringer solution. A reduction of 30-day postoperative SSI I–III rates by lavage with NaOCl/HOCl solution is postulated. Since there is currently no data on incidence rates from randomized clinical trials, the rates for sample size calculation were estimated according to the clinical experience at our institution. Therefore, the study is designed with one unblinded look at the data, which will be performed after half of the patients reached the primary endpoint (30 days post-surgery), by a second study statistician, who will not participate in the final study analysis. This interim information will be used to check the assumptions which were used to calculate the sample size for the study. If needed, the sample size will be adjusted.

## Methods: participants, interventions, and outcomes

### Study setting {9}

The PAISI study will be conducted in the surgical department of the “Klinikum Rechts der Isar” of the Technical University of Munich, which is a member of the German trial network (CHIR-*Net*) of the German Surgical Society (*Deutsche Gesellschaft für Chirurgie*) and has extensive experience regarding studies on prevention of SSIs. All study personnel involved has adequate training and will be instructed in all specific procedures before the initiation of the study. The leading surgeon of the operating team will perform the intervention since it represents a routine technique. All participating surgeons will be instructed and authorized by the investigator, prior to the first study procedure.

### Eligibility criteria {10}

#### Inclusion criteria

- Clean-contaminated, contaminated, or dirty surgery (LOC II–IV) according to CDC classification (Table 2)
- Elective and emergency surgery by midline or transverse laparotomy for hepatobiliary or gastrointestinal resections
- Age ≥ 18 years
- American Society of Anaesthesiologists (ASA) score ≤ 3
- Ability to understand the nature and extent of the study and to give written informed consent.

**Table 2:** Levels of contamination (LOC) adapted from CDC and the corresponding SSI rates [6, 7]

LOC	Criteria	SSI rates
Class I/clean	- Uninfected operative wounds - No inflammation is encountered - The respiratory, alimentary, genital, or uninfected urinary tracts are **not** entered.	1–5%
Class II/clean-contaminated	- The respiratory, alimentary, genital, or urinary tracts are **entered under controlled conditions and without unusual contamination**. - No evidence of infection or major break in technique is encountered.	3–11%
Class III/contaminated	- Operations with **major breaks in sterile technique or gross spillage from the gastrointestinal tract** - Incisions in which **acute, non-purulent inflammation** is encountered - Outside object had contact with the wound (e.g. a bullet, knife blade).	10–17%
Class IV/dirty-infected	- Involve existing clinical infection - Perforated viscera - Foreign object lodged in the wound - Any wound that has been exposed to pus or faecal matter.	>27%

#### Exclusion criteria

- Inability to give/understand informed consent
- ASA > 3
- Incompliance to adhere to the follow-up visit schedule or telephone interview
- Clean procedures (LOC I) according to the CDC classification or surgery without opening of the abdominal cavity
- Laparoscopic surgery
- Revision-surgery (previous abdominal surgery within the last 30 days)
- Planned re-laparotomy within 30 days
- Severe immunosuppression
- Terminal kidney failure requiring dialysis
- Concurrent abdominal wall infections
- Participation in another clinical trial that interferes with the primary or secondary outcomes of this study.

To enhance generalizability and representativeness, all patients undergoing elective and emergency laparotomy (transverse or midline) for hepatobiliary or gastrointestinal surgery will be screened. However, only clean-contaminated, contaminated or dirty (LOC II–IV), open abdominal surgery, according to the CDC classification [6] will be eligible, since in clean (LOC I) procedures the risk of SSI is generally low (see Table 2). Laparoscopic surgery as well as surgery without opening of the abdominal cavity or revision surgery (previous abdominal surgery within the last 30 days or planned re-laparotomy within the next 30 days of surgery) will be excluded, since these types of procedures are not comparable in terms of SSI risk.

Patients have to be ≥ 18 years of age and able to understand and give written informed consent. Any patient in a critical general medical condition (ASA > 3) will be excluded to avoid too many patient-related confounders. Furthermore, patients must be able and willing to attend follow-up visits. Patients with severe immunosuppression (e.g. after: organ or bone marrow transplantation, concurrent steroid treatment with >10 mg prednisone daily or an equivalent dose of any other steroid), concurrent infliximab treatment or treatment with an equivalent immunosuppressive substance or chemotherapy within the last 2 weeks prior to study intervention) or patients with severe pre-operative neutropenia (≤ 0.5 × 10^9^/L) or liver cirrhosis Child-Pugh B/C will not be included, since they differ in terms of SSI risk. Furthermore, patients with terminal kidney failure depending on dialysis treatment will not be included as it is not known if the intraabdominal irrigation with electrolyte solutions might further disturb the serum electrolyte levels. Lastly, patients that participate in other clinical trials that could interfere with the primary or secondary outcomes of the PAISI study will be excluded.

### Who will take informed consent? {26a}

A patient can only be included in the study, if he provides written consent after being informed by a clinical investigator (orally and in writing) about the nature, significance, and scope of the clinical study in an appropriate and understandable way. Clinical investigators are specifically trained medical doctors of the local study team. The investigator must fully explain the purpose of the study to the patient or his/her guardian prior to entering the patient into the study. The investigator is responsible for obtaining written informed consent from each patient.

### Additional consent provisions for collection and use of participant data and biological specimens {26b}

Not applicable as no biological specimens are collected, and the participant data will not be used for other purposes than the study. The IC form contains a data protection statement regarding the consent of patient must be given that relevant data can be used for the study in a pseudonymous form.

## Interventions

### Explanation for the choice of comparators {6b}

The IPI with physiological electrolyte solution (e.g. Ringer’s solution) at the end of abdominal surgical procedures represents a current clinical routine procedure. In many hospitals, even IPI with NaOCl/HOCl (50/50ppm) solutions is already considered the gold standard for septic or contaminated procedures. However, most hospitals do not have standard protocols but leave the decision if, how and when to irrigate the abdominal cavity and/or the incisional wound up to the surgeon. However, in current national and international guidelines, there are no specific recommendations on the performance, volume or timing of IPI [5]. Modern antiseptic solutions like NaOCl/HOCl or Polyhexanide are not mentioned in the current guidelines.

No additional risks for study patients are anticipated since IPI represents a clinically established standard method. NaOCl/HOCl (50/50ppm) and physiological Ringer’s solution are CE-certified for peritoneal and surgical wound irrigation of soft tissue wounds. The study will be planned, conducted, and analysed according to all relevant rules and regulations and the Declaration of Helsinki, 2008. No specific risks are expected because neither application of NaOCl/HOCl nor Ringer’s solution will have systemic effects on the participants. Adverse effects may only be expected in the improbable event of accidental contamination of the respective irrigation solutions. The potential benefits of reduced SSIs outweigh the mentioned negligible potential adverse effects. The informed consent process adheres to Good Scientific Practice, which maximizes patients’ safety and confidentiality.

### Intervention description {11a}

At the end of surgery, before the closure of the abdominal fascia, patients will be randomized stratified by LOC of the operation. In the experimental group, the abdominal cavity will be irrigated with 1000 ml NaOCl/HOCl (50/50ppm) solution. In addition, after closure of the fascia, the wound shall be carefully rinsed throughout with another 500 ml of the irrigation solution and the excess removed with suction. Debris and blood clots should be removed from the wound using irrigation/suction. The wound shall not be rubbed dry with abdominal cloths but left moistened with the irrigation solution to ensure the desired antiseptic effect. After irrigation with NaOCl/HOCl, the abdominal cavity and wound shall not be irrigated with any other solution again.

In the control group, the same intervention will be performed using 1000 ml of Ringer’s solution for the abdominal lavage and 500 ml of the same solution for the incisional wound (current standard). The irrigation volume of 1500 ml was chosen to be sure that the abdominal cavity and even large laparotomy wounds would be sufficiently irrigated. This was determined by senior surgeons´ clinical experience, since so far, no recommendations for the optimal volume of surgical irrigation exist. Closure of the fascia and the skin will be performed according to local standards, without any further wound-related procedure.

NaOCl/HOCl (50/50ppm) and Ringer’s solutions are purchased, stored, and distributed according to the local standard operating procedures. Trade name, dosage, batch and dispensed amount will be documented on a separate form.

### Criteria for discontinuing or modifying allocated interventions {11b}

Participants of the study can withdraw their consent to take part at any time without declaration of reasons. All hitherto collected data are subject to analysis, within the consent of the participant. The principal investigator may exclude patients from the study, if patients’ safety is at risk or if there is insufficient compliance of the patient. In order to generate a meaningful database, excluded patients can be replaced by recruitment of new patients. If a patient does not receive NaOCl/HOCl or Ringer solution irrigation of the peritoneal cavity or wound, this does not automatically lead to exclusion of the study.

### Strategies to improve adherence to interventions {11c}

Not applicable as the intervention is performed only once, intraoperatively. Adherence to the follow-up visit schedule is promoted by facilitating the last two study visits 10 and 30 days postoperatively as a structured telephone interview. Furthermore, all study procedures and visits closely orientate towards standard medical care.

### Relevant concomitant care permitted or prohibited during the trial {11d}

No additional treatments or interventions will be performed within the study. All patients will receive standard perioperative antibiotic prophylaxis, the timing and type of which will be recorded in the eCRF. Furthermore, the application of abdominal wall protectors is recommended for contaminated procedures and must be recorded in the eCRF. A change of gloves ahead of wound closure is frequently performed in contaminated procedures and must also be recorded in the eCRF. If an irrigation-suction-drain is placed in the abdominal cavity and the patient receives prophylactic intraabdominal irrigation therapy via this device postoperatively, the patient must be excluded from the study. If indicated for medical reasons, all kind of medication is permitted during the study. Postoperative medication with known adverse effects on wound healing (e.g. antibiotics, corticoids, and other immunosuppressive agents) will be recorded in the eCRF. Any operative or interventional revision of the wound will be documented as AE/ SAE in the eCRF.

### Provisions for post-trial care {30}

Not applicable as no additional risks and harms from study participation are anticipated, and no additional travel is necessary for the patient.

### Outcomes {12}

The primary efficacy endpoint of the study is occurrence of SSI within 30 days postoperatively, according to the internationally accepted and recommended SSI definition by the CDC (Table 1) [6]. This endpoint has been used in previous studies and assures comparability of the results [8–10, 25]. This endpoint is further considered to be of clinical relevance as SSI increases morbidity and mortality of individual patients, direct and indirect costs and prolongs hospital stay as outlined before. The duration of hospital stay is considered as a secondary outcome, in order to evaluate the potential economic benefit of the intervention. In addition, the following outcome measures have been defined as secondary endpoint measures and will be determined by the unit given in parentheses:

- Rate of SSI by CDC class I–III (superficial, deep, organ-space) (%)
- Duration of hospital stay up to 30 days after surgery (days);
- 30 days mortality (%);
- 30 days rate of reoperation/relaparotomy (%);
- 30 days rate of postoperative complications in both groups (%). All AE/SAEs will be additionally classified according to the Clavien-Dindo classification of surgical complications and the Comprehensive Complication Index [26, 27].

### Participant timeline {13}

**Fig. 1:**
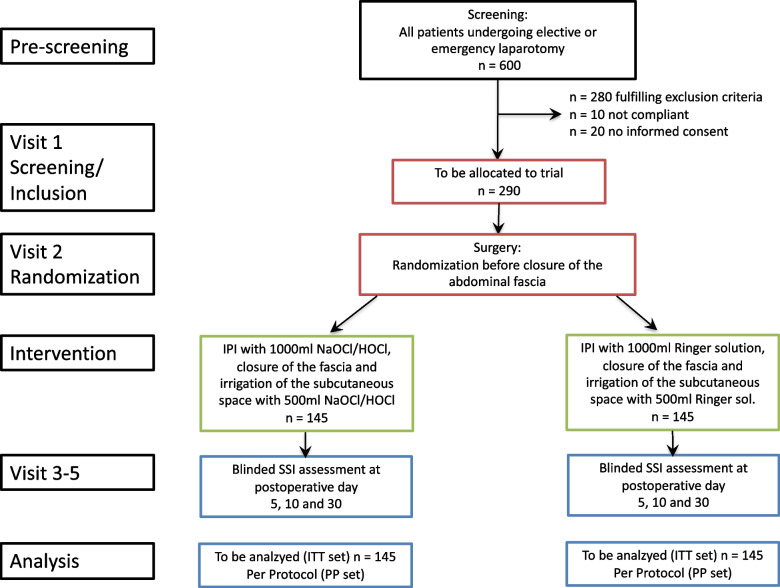
PAISI intervention flow scheme (according to SPIRIT statement 2013 [28])

Figure 1 and Table 3 reflect the intervention scheme flow and visits for the PAISI study. Visits are the same for all participants of the study, regardless of the intervention group.

**Table 3:** PAISI study visits (according to SPIRIT statement 2013 [28])

	Study period				
	Inclu.	Rand.	Post-allocation		Close-out
Study visit Number	1	2	3	4	5
Timepoint	− 1–3 days	day 0)	day 5	day 10 (+5)	day 30 (+5)
Informed consent	X				
Inclusion and exclusion criteria	X				
Randomization		X			
Intervention (IPI with 1500ml NaOCl/HOCl solution)		X			
Control (IPI with 1500ml Ringer solution)		X			
Demographical data	X				
Medical history and Charlson Comorbidity Index	X				
Medication with known effects on wound healing	X				
Physical examination	X				
Type of operation		X			
Duration of operation		X			
Level of contamination		X			
Type and length of incision		X			
Abdominal wall closure technique and suture material		X			
Presence of an enterostomy		X			
Use of wound edge protectors		X			
Changing of gloves		X			
Placement and type of-intra-abd. drains		X			
Postoperative medication with known effects on wound healing			X	X	X
Documentation of SSI			X	X	X
Documentation of other postoperative complications (Clavien Dindo and CCI)			X	X	X
Documentation of re-operation			X	X	X
Documentation of postoperative intra-abd. irrigation via irrigation-suction drainage system			X	X	X
Documentation of AE/SAE of special interest		X	X	X	X
Duration of hospital stay					X

### Sample size {14}

The sample size was calculated (nQuery 8 software, version 8.6.1.0, Statistical Solutions Ltd, Cork, Ireland) based on the primary endpoint of the study. Assuming SSI rates of 4% and 16% for the experimental and the standard group respectively (OR=4.571), the one-sided z-test (Group Sequential test of two proportions) at the 2.5% significance level with a total of two looks at the data will have overall 80% power to show superiority of the experimental over the standard arm if the study includes at least 232 patients (116 patients per intervention group). The calculation is continuity corrected due to the expected low proportion in the experimental group. The results assume that the group sequential design has one interim sequential test for monitoring and sample size re-estimation (two total looks at the data including final analysis), which means that an interim analysis is planned after half of the patients have reached 30 days post-surgery. The O’Brien-Fleming spending function is used to determine the efficacy test boundary and the non-binding futility boundary. The group sequential test boundaries (Z scale) for the interim and the final analysis are as follows:

Upper efficacy bounds: 2.963 and 1.969

Futility bounds: 0.555 (non-binding) and 1.1969.

A drop-out rate of about 20% is expected in this study, based on experience from similar trials previously conducted within the CHIR-*Net* trial network. Therefore, a total of 290 patients (145 per intervention group) should be included in the study.

### Recruitment {15}

The recruitment period is expected to be approximately 32 months (first patient in, to last patient out 33 months). In the study site, an average of around 80 laparotomies LOC II–IV are performed per month. Experience from previous clinical studies shows that the recruitment of 10 patients per month is feasible here. To cover any unforeseen recruitment difficulties, for example due to the COVID-19 pandemic, 3 extra months of recruitment time were added. In the case of elective procedures, pre-screening (this is just a pre-selection of eligible patients within the study team) of patients can be performed up to 14 days prior to the scheduled surgical procedure. In case of emergency procedures, screening and inclusion can take place on the day of admission to the hospital, which is usually the same day as surgery.

## Assignment of interventions: allocation

### Sequence generation {16a}

The randomization list will be generated by an online randomization tool (Randomizer, Institute for Medical Informatics, Statistics and Documentation, Medical University of Graz, Austria; URL: http://www.randomizer.at). Online-Randomization will be performed at the end of the surgical procedure before the abdominal fascia is closed and will be stratified by LOC of the surgical procedure (LOC II–IV; Table 2). To assure balanced group sizes during the accrual, a block-wise randomization is applied.

### Concealment mechanism {16b}

Only designated members of the study team, who will not perform postoperative study visits, will have access to the online randomization tool. After informing the surgical team of the randomization result, the randomization sheets will be printed out and must be stored away from the patient records, study documents and ISF to ensure blinding of the rest of the local study team.

### Implementation {16c}

The allocation sequence is generated by the Randomizer tool (Institute for Medical Informatics, Statistics and Documentation, Medical University of Graz, Austria; URL: http://www.randomizer.at). Participants will be enrolled by the specifically trained medical doctors of the local study team. Participants will be assigned to the intervention groups by the designated study nurse or medical doctor who is effectuating the online randomization.

## Assignment of interventions: blinding

### Who will be blinded {17a}

The blinding procedure is restricted to participating patients, the statistician who performs the final analysis and the outcome assessors. Blinding of the surgical team that performs the intervention is impossible because of the distinctive smell of NaOCl/HOCl solution. Postoperatively, an outcome assessor of the local study group, who is unaware of the patient’s intraoperative intervention, will clinically assess the primary endpoint (SSI) on 3 postoperative study visits.

### Procedure for unblinding if needed {17b}

Unblinding of outcome assessors is permissible if significant hazards for patients’ safety or welfare occur. In such an unlikely case, the member of the study team responsible for the randomization procedure can unblind the outcome assessor for the randomization result of the concerned patient, only.

## Data collection and management

### Plans for assessment and collection of outcomes {18a}

#### Screening visit

Screening and inclusion of patients will be performed not earlier than 3 days and not later than one day before the planned surgical procedure, to ensure the patient has enough time to consider the decision to participate in the study. All screened patients are documented in a screening log. If patients do not wish to participate in the study, the reasons will be documented accordingly. If patients fit inclusion/exclusion criteria and agree to participate, they will need to give written informed consent, after adequate time for consideration. Therefore, at the screening visit, a detailed description of the study and further instructions are discussed with the patient, including methods of abdominal and wound irrigation, risk-benefit ratio, and follow-up schedule.

#### Visit 1 (inclusion)

After the investigator has reviewed the inclusion and exclusion criteria again and having received written consent by a patient, demographical data and medical history (incl. age, gender, body height, body weight, BMI, ASA, concurrent medication with effects on wound healing, history of SSI, history of radio/chemotherapy, diabetes, smoking, alcohol consumption, duration of pre-operative hospital stay) and the diagnosis will be documented. In addition, the Charlson Comorbidity Index (CCI) will be calculated, which is a prognostic score that is used in many recent trials to simplify the comparability and evaluation of multimorbid study populations [29]. In addition, the investigator will perform a physical exam of the planned abdominal incision area.

#### Visit 2 (surgery/randomization)

Documented parameters of the surgical procedure include the urgency (emergency/elective), type of surgical procedure (colorectal and/or small bowel and/or hepato-biliary and/or pancreatic and/or splenectomy and/or gastric and/or oesophageal and/or others) the duration of surgery (incision until complete skin closure), the LOC according to CDC classification (LOC II–IV; see Table 2), the type and timing of perioperative antibiotic prophylaxis, the intraoperative use of wound edge protectors, and the prophylactic changing of gloves during of the operation. The type (transverse/midline) and length (cm) of the incision, as well as the creation or presence of an enterostomy (yes/no), the abdominal wall and wound closure technique (subcutaneous sutures, stapler/suture, continuous/single) and used suture material, the placement and type of intraabdominal drains will also be recorded. If the operating surgeon decides that incomplete closure of the wound and/or any other wound-related procedure after the study intervention (e.g. negative pressure treatment) is necessary for the benefit of the patient, the patient will have to be excluded from the study. Furthermore, if prophylactic or therapeutic postoperative intraabdominal irrigation is performed via an irrigation-suction-drain system, the patient must be excluded from the study.

#### Visit 3 to 5 (post-op days 5, 10, and 30)

Postoperatively, there will be 3 study visits where an independent, blinded outcome assessor trained in the diagnosis and classification of SSI according to CDC definition (SSI I–III, see Table 1) will examine wounds. Postoperative medication with known effects on wound healing (e.g. antibiotics, corticoids and other immunosuppressive agents) will be documented in the eCRF. Any surgical complication, including SSI, will be reported including its severity (according to the Clavien-Dindo Classification and CCI) and its consequent treatment. Furthermore, the rate of re-operations, mortality and occurrence of any AE or SAE of special interest will be documented. Additionally, the duration of the hospital stay (in days) will be documented on visit 5 (post-op day 30).

### Plans to promote participant retention and complete follow-up {18b}

To promote complete follow-up, a visit window of two additional days was implemented for visit 3, and 5 additional days for visits 4 and 5. If however, the patient is unable to attend visit 4 or 5 due to postoperative treatment in a rehabilitation facility or other medical reasons, a standardized protocol for evaluation and documentation of the wound or intraabdominal complications will be sent to and filled out by the treating physician. In the exceptional case that the patient has no possibility to be evaluated by a physician for visit 5, a telephone interview with the patient can be performed to assess the primary endpoint. However, in this case, the reasons why a physician cannot see the patient have to be declared on a designated form.

### Data management {19}

The documentation of the study data in adherence to the protocol is the responsibility of the principal investigator. Original data (source documents) remain in hospital medical record and information on the eCRF must be traceable and consistent with the original data. Original written informed consent signed by the patient is kept by the investigator and a signed copy will be given to the patient. No information in source documents about the identity of the patients will be disclosed. All data collected in this study must be entered in an eCRF using a REDCap database (Research Electronic Data Capture, Version 10.5.2, 2022, Vanderbilt University) [30, 31] which has to be completed by the investigator or authorized study personnel and signed by the investigator. This also applies for those patients who do not complete the study. If a patient is excluded from the study, the reason must be recorded on the eCRF. The investigator is responsible for ensuring the accuracy, completeness, and timeliness of all data in the eCRFs and in all required reports. After entry of all collected data and clarification of all queries, the database will be closed at the completion of the study. Data and results electronically recorded will be archived according to applicable law.

### Confidentiality {27}

The applicable local regulations of data privacy protection will be followed. The patients will be informed that any patient-related data and materials will be appropriately made pseudonymous and that these data may be used for analysis and publication purposes. Furthermore, the patients will be informed that their data may be inspected by monitors for the purpose of validation of a proper study conduct. Patients who do not provide consent for processing of their data, according to the data protection agreement included in the ICF, will not be included in the clinical study.

### Plans for collection, laboratory evaluation and storage of biological specimens for genetic or molecular analysis in this trial/future use {33}

Not applicable since no biological specimen will be taken.

## Statistical methods

### Statistical methods for primary and secondary outcomes {20a}

The intention-to-treat (ITT) set consists of all patients who received irrigation of the abdominal cavity before closure of the abdominal fascia with one of the study solutions. Analysis will be performed as randomized regardless of the actual intervention and regardless of any further medical or surgical treatments. The Per-Protocol (PP) set consists of patients treated according to protocol, excluding any major protocol violations. The PP set will be analysed as treated. The Safety (SA) set will consist of all patients in the ITT set, analysed as treated.

All primary and secondary analyses will be performed on the ITT set. Sensitivity analysis of the primary endpoint will be performed on the PP set. The SA set will be used for the safety analyses. The incidence of SSI within 30 days after surgery will be compared between the two study groups using the one-sided group sequential test of two proportions at the 2.5% significance level with a total of two looks at the data. The primary endpoint analysis will be performed on the ITT set. Since the ITT set includes all patients who were treated in the study regardless of any further medical or surgical treatments, it is expected, that the SSI rates 30 days post-surgery will be available for most patients. Nevertheless, it is possible that some patients do not reach the primary endpoint due to death, withdrawal of informed consent, loss to follow-up, or other unforeseen reasons. Patients who die before day 30 will be considered as having SSI at time of death regardless of the intervention group, unless SSI was documented earlier. All other missing primary endpoint data will be dealt with using multiple imputations based on binary logistic regression models including LOC, ASA, BMI, age, diabetes, type of surgery, duration of surgery, use of wound-edge protectors, and intraoperative changing of gloves.

### Interim analyses {21b}

Thirty days after the first half of the patients has reached the primary endpoint, an interim analysis will be performed by a second statistician, who does not take part in the final study analysis. A decision will be made by the study leadership, using the blinded results to stop the study with proven efficacy, stop the study for futility, continue the study as planned, or continue the study after adjusting the sample size.

### Methods for additional analyses (e.g. subgroup analyses) {20b}

Since randomization will be stratified by LOC, supportive analysis of the primary endpoint will be performed using a binary logistic regression model with dependent variable SSI and covariates intervention group and LOC. In addition, the following parameters might influence the outcome, which is why they will be included as model covariates in an additional model:

- Operation-related risk factors: (a) type of surgery; (b) duration of surgery; (c) NNIS Risk Score, (d) use of wound-edge protectors; (e) intraoperative changing of gloves before skin closure; (f) presence of an enterostomy; (g) administration and timing of antibiotic prophylaxis;
- Patient-related risk factors: (g) BMI; (h) ASA score; (i) Age; (j) diabetes; (k) smoking; (l) alcohol abuse; (m) Charlson Comorbidity Index, (n) history of SSI; (o) history of radiotherapy; (p) history of chemotherapy, (q) days of hospitalization prior to surgery.

Supportive analysis of the primary endpoint will be performed on the ITT set using multiple imputations for any missing SSI values and on the PP set. Secondary endpoints will be analysed by intervention arm on the ITT set, using appropriate descriptive statistics. Any explorative statistical testing will be performed using a significance level of 5%. The assessment of safety will be based on the frequency of AE/SAE as defined in the protocol within the safety population consisting of all patients randomized into the study.

### Methods in analysis to handle protocol non-adherence and any statistical methods to handle missing data {20c}

Multiple imputations will be used for missing primary endpoint data. All other data will be analysed on a complete-case basis.

### Plans to give access to the full protocol, participant level-data and statistical code {31c}

The protocol publication will be accessible on the DRKS website (www.drks.de). This includes a summary in lay language for the interested patient community. The full protocol and statistical code can be requested from the authors. Individual participant level data that underline the results reported in this article can be shared after anonymization, with investigators whose proposed use for the data has been approved by an independent review committee identified for this purpose. Proposals can be submitted up to 36 months after publication of the study results.

## Oversight and monitoring

### Composition of the coordinating Centre and trial steering committee {5d}

As this study is planned as a monocentric trial, there is no coordinating centre. The trial steering committee in this setting consist of the study leadership (principal investigator, deputy investigator and coordinating investigator) which is responsible for recruitment of patients and data management. The statistical analysis will be performed by an independent statistician. Trained medical doctors of the local study group will take informed consent. The study leadership will meet once a month to discuss progress and problems regarding the trial. Following the planned interim analysis after 50% of patients are recruited a meeting with the trial statistician and the scientific advisors as well as the funders will be held to discuss the results and consequences. The ethics committee will be informed about the outcome of this meeting. Public involvement is not planned in this study.

### Composition of the data monitoring committee, its role and reporting structure {21a}

In this study setting, a data monitoring committee is not planned, as the intervention poses no additional risks for the patients and is a routine medical procedure.

### Adverse event (AE) reporting and harms {22}

Each adverse event (AE) is to be classified by the investigator according to the Common Terminology Criteria CTCAE V 5.0 [32]. Serious AEs and AEs of special interest are captured and processed until the last visit of the last patient. A final list will be provided to the ethics committee. All documented (S)AEs should be followed to resolution or stabilization. The investigator will be requested to supply as much detailed information as possible regarding the event that is available at the time of the initial contact. The investigator is also required to complete missing or requested information until the (S)AE has resolved or, in the case of permanent impairment, until stabilized. All (S)AEs severity will be additionally graded by the Clavien Dindo Classification of postoperative complications and the CCI.

### Frequency and plans for auditing trial conduct {23}

As this study is comparing two routine surgical procedures, auditing of the trial by external institutions is not required and therefore not planned.

### Plans for communicating important protocol amendments to relevant parties (e.g. trial participants, ethical committees) {25}

Any amendments to the protocol, other than administrative ones (of which the ethics committee will merely be informed), must be reviewed and approved by the ethics committee. The changes will be updated in the clinical register DRKS on a regular basis. If necessary, patient information is updated to inform new participants of the changes.

### Dissemination plans {31a}

After completion of the clinical study, a manuscript of the study results will be prepared for publication in a reputable scientific journal according to the CONSORT statement. For this manuscript, final analyses will be generated from the study database, and it will be subject to review by the principal investigator. The use of professional writers is not intended.

## Discussion

SSI remain one of the most common complications following abdominal visceral surgery (14–25%) [8–10, 25] and dramatically increase the length of hospital stay and treatment costs. Hypothetically, antiseptic IPI at the end of surgery seems to be a pragmatic measure to reduce SSI rates, especially organ/space infections (SSI class III). Currently, the official recommendations on the prophylactic use of IPI and clinical practice vary largely. The existing evidence is low level and does not solely focus on visceral surgery but includes all types of surgery (e.g. orthopaedic- or neurosurgery), which differ substantially in SSI rates and causative microorganisms. However, despite its unproven efficacy, most general and visceral surgeons currently use IPI to prevent SSI. The PAISI study has a pragmatic, 2-armed study design (NaOCl/HOCl 50/50ppm *vs.* Ringer intraoperative peritoneal and wound irrigation). Internal validity and data quality assurance are established by adherence to the SPIRIT guideline regarding recruitment, methods against bias, outcome reporting and documentation. All patients undergoing gastrointestinal or hepatobiliary surgery (LOC II–IV) by laparotomy within 32 months will be screened for this study. Broad inclusion criteria were applied to ensure rapid and sufficient recruitment of the target sample size. The primary endpoint is the incidence of SSI 30 days postoperatively, according to the CDC definition and classification (Table 1) [6]. Since many different SSI definitions have been proposed in the past decades, standardized reporting is crucial for the comparability of studies regarding SSI prevention. The results of this pragmatic study will provide evidence for clinical recommendations regarding the use of antiseptic IPI with NaOCl/HOCl solution to prevent SSI after laparotomy and potentially impact future clinical guidelines. Furthermore, the results will provide a base for future research and provide participating patients the opportunity of an improved treatment.

### Trial status

The first patient was recruited in September 2022, therefore recruitment is planned to be completed approximately by May 2025.

## Data Availability

After completion of the study, individual participant data that underline the results reported in the published article can be shared after anonymization with investigators whose proposed use for the data has been approved by an independent review committee identified for this purpose. Proposals can be submitted up to 36 months after publication of the study results.

## Abbreviations

AE: Adverse event
ASA: American Society of Anaesthesiologists
BMI: Body mass index
CDC: Centre for Disease Control and Prevention
CI: Confidence interval
CTCAE: Common Terminology Criteria for Adverse Events
DRKS: Deutsches Register Klinischer Studien
eCRF: Electronic case report form
ECG: Electrocardiogram
EDTA: Ethylene-diamineteraacetic acid
HOCl: Hypochlorite
ICF: Informed consent form
ICTRP: International Clinical Trials Registry Platform
IPI: Intraperitoneal irrigation
ISF: Investigator site file
ITT: Intention-to-treat
K: Potassium
LOC: Level of contamination
MRI: Klinikum München rechts der Isar
MSZ: Münchner Studienzentrum
Na: Sodium
NaCl: Sodium chlorite
NaOCl: Sodium hypochlorite
PHX: Polyhexanide
PP: Per protocol
PVP-I: Polyvinylpyrrolidone, povidone-iodine
RCT: Randomized controlled trial
SAE: Serious adverse event
SAS: Statistical analysis system
SSI: Surgical site infection
TUM: Technical University of Munich
WHO: World Health Organization
